# Role of transient receptor potential ankyrin 1 channels in Alzheimer’s disease

**DOI:** 10.1186/s12974-016-0557-z

**Published:** 2016-04-27

**Authors:** Kuan-I Lee, Hsueh-Te Lee, Hui-Ching Lin, Huey-Jen Tsay, Feng-Chuan Tsai, Song-Kun Shyue, Tzong-Shyuan Lee

**Affiliations:** Department of Physiology, School of Medicine, National Yang-Ming University, Taipei, Taiwan; Institute of Anatomy and Cell Biology, National Yang-Ming University, Taipei, Taiwan; Institute of Neuroscience, School of Life Science, National Yang-Ming University, Taipei, Taiwan; International Graduate Program, Interdisciplinary Neuroscience Program, Taipei, Taiwan; Cardiovascular Division, Institute of Biomedical Sciences, Academia Sinica, Taipei, Taiwan; Genome Research Center, National Yang-Ming University, Taipei, Taiwan

**Keywords:** Transient receptor potential ankyrin 1, Calcium, Alzheimer’s disease, Inflammation, Protein phosphatase 2B

## Abstract

**Background:**

Transient receptor potential ankyrin 1 (TRPA1) channel plays an important role in pain and inflammation. However, little is known about the significance of the TRPA1 channel in the pathophysiology of Alzheimer’s disease (AD).

**Methods:**

Wild-type (WT), TRPA1^−/−^, amyloid precursor protein (APP)/presenilin 1 (PS1) transgenic (APP/PS1 Tg) mice, the mouse model of AD, and APP/PS1 Tg/TRPA1^−/−^ mice were used to examine the role of TRPA1 in pathogenesis of AD. Western blot was used for protein expression; immunohistochemistry was used for histological examination. The mouse behaviors were evaluated by locomotion, nesting building, Y-maze and Morris water maze tests; levels of interleukin (IL)-1β, IL-4, IL-6 and IL-10 and the activities of protein phosphatase 2B (PP2B), NF-κB and nuclear factor of activated T cells (NFAT) were measured by conventional assay kits; Fluo-8 NW calcium (Ca^2+^) assay kit was used for the measurement of intracellular Ca^2+^ level in primary astrocytes and HEK293 cells.

**Results:**

The protein expression of TRPA1 channels was higher in brains, mainly astrocytes of the hippocampus, from APP/PS1 Tg mice than WT mice. Ablation of TRPA1-channel function in APP/PS1 Tg mice alleviated behavioral dysfunction, Aβ plaque deposition and pro-inflammatory cytokine production but increased astrogliosis in brain lesions. TRPA1 channels were activated and Ca^2+^ influx was elicited in both astrocytes and TRPA1-transfected HEK293 cells treated with fibrilized Aβ_1–42_; these were abrogated by pharmacological inhibition of TRPA1 channel activity, disruption of TRPA1 channel function or removal of extracellular Ca^2+^. Inhibition of TRPA1 channel activity exacerbated Aβ_1–42_–induced astrogliosis but inhibited Aβ_1–42_–increased PP2B activation, the production of pro-inflammatory cytokines and activities of transcriptional factors NF-κB and NFAT in astrocytes and in APP/PS1 Tg mice. Pharmacological inhibition of PP2B activity diminished the fibrilized Aβ_1–42_–induced production of pro-inflammatory cytokines, activation of NF-κB and NFAT and astrogliosis in astrocytes.

**Conclusions:**

TRPA1 − Ca^2+^ − PP2B signaling may play a crucial role in regulating astrocyte-derived inflammation and pathogenesis of AD.

**Electronic supplementary material:**

The online version of this article (doi:10.1186/s12974-016-0557-z) contains supplementary material, which is available to authorized users.

## Background

Alzheimer's disease (AD), a progressive neurodegenerative disease, is the most common cause of dementia [1]. The typical symptoms of AD include learning and memory loss and cognitive impairment [1, 2]. Amyloid-β (Aβ) plaque deposition is one of the major pathological hallmarks in AD [2–4]. Aβ is generated from amyloid precursor protein (APP) by β-site APP cleavage enzyme 1 (BACE1) and presenilin 1 (PS1) [3, 5]. The toxic Aβ activates astrocytes and microglia to trigger chronic inflammation, thereby releasing cytokines, pro-inflammatory mediators and reactive oxygen species (ROS) and leading to progression of AD [6–10]. Several lines of evidence suggest that Aβ-mobilized calcium (Ca^2+^) flow is closely associated with the inflammatory response in astrocytes [11–13]. On Aβ stimulation, elevated intracellular Ca^2+^ level promotes cytokine secretion by activating protein phosphatase 2B (PP2B, also called calcineurin) and its downstream NF-κB and nuclear factor of activated T cells (NFAT) [14–16]. However, which type of cation channel is responsible for the Aβ-activated Ca^2+^ signaling and inflammation remains for further investigation.

Transient receptor potential ankyrin 1 (TRPA1) channel is a type of nonselective transmembrane cation channel with multiple ankyrin repeats on its N-terminal [17–20]. TRPA1 channel is expressed in primary sensory neurons and non-neuronal cells and may be a sensor for detecting ROS, cold temperature and cannabinoids [21–24]. Upon detection of these signals, the TRPA1 channel is activated, which results in increased intracellular Ca^2+^ levels and activated downstream signaling cascades [17–20]. In the brain, the TRPA1 channel plays an important role in regulating brain development and the physiological function of astrocytes [24, 25]. In addition, it may be a key gatekeeper in regulating the inflammatory response with stimuli including bacterial endotoxin, environmental irritants or inflammatory mediators [26–28]. Recently, AD research has focused on the emerging role of Ca^2+^-related signaling pathways in the pathogenesis of AD [29, 30]. However, the role and underlying molecular mechanism(s) of the TRPA1–Ca^2+^ signaling cascade in AD pathogenesis are still elusive.

In this study, we aimed to investigate the role of TRPA1 channels in AD pathogenesis and the possible molecular mechanisms in a mouse model. We determined the expression of TRPA1 channels *in vivo* by using wild-type (WT) and APP/PS1 transgenic (Tg) mice, then investigated whether the TRPA1 channel plays a role in the development of AD by using APP/PS1 Tg and APP/PS1 Tg/TRPA1^−/−^ mice. Finally, we assessed the importance of TRPA1 and the potential mechanism underlying the regulation of Aβ-mediated inflammation in mice and astrocytes.

## Methods

### Reagents

Rabbit antibody for TRPA1 (NB110-40763) was from Novus (Littleton, CO, USA). Goat antibody for Akt (sc-1619), rabbit antibodies for PP2B (sc-9070), mouse antibodies for glial fibrillary acidic protein (GFAP, sc-166481), goat anti-rabbit FITC-conjugated (sc-2012), goat anti- mouse Texas red-conjugated (sc-2781) and FITC-conjugated (sc-2010) antibodies were from Santa Cruz Biotechnology (Santa Cruz, CA, USA). Mouse antibodies for GFAP (MAB360), NeuN (MAB3770), ionized calcium-binding adapter molecule 1 (IBA-1, MABN92), von Willebrand factor (vWF, MAB7356), and the cellular PP2B activity kit were from Millipore (Darmstadt, Germany). Rabbit LDLR-related protein 1 (LRP-1, L2170), mouse antibody for α-tubulin (T-9026), bovine serum albumin (BSA), phosphatase inhibitor cocktails 1 and 2, HC030031, allyl isothiocyanate (AITC), ethylene glycol tetraaceticacid (EGTA), ethylenediaminetetraacetic acid (EDTA), cyclosporine (CsA) and fenvalerate (Fen) were from Sigma-Aldrich (St. Louis, MO, USA). Mouse antibody for Aβ (SIG-39320-200) was from Covance (Dedham, MA, USA). Rabbit antibody for β-APP C-terminal fragment (βCTF, 802801) was from BioLegend (San Diego, CA, USA). Mouse antibody for ATP-binding cassette transporter A1 (ABCA1, ab18180), IL-4 (ab9622) and IL-10 (ab9969) were from Abcam (Cambridge, MA, UK). Rabbit antibody for apolipoprotein E (apoE, 1930-5) was from Epitomics (Burlingame, CA, USA). Mouse anti-phosphor-Akt (587 F11) was from Cell Signaling (Danvers, MA, USA). Retrieval buffer was from Biocare Medical (Concord, CA, USA). The mounting medium with DAPI was from Vector Laboratories (Burlingame, CA, USA). TurboFect was from Fermentas (Glen Burnie, MD, USA). The ELISA kit for NF-κB activity was from Cayman Chemical (Ann Arbor, MI, USA) and for NFAT activity was from Active Motif (Carlsbad, CA, USA). ELISA kits for IL-1β, IL-4, IL-6 and IL-10 and mouse antibodies for IL-1β (AF-401-NA) and IL-10 (AF-406-NA) were from R&D systems (Minneapolis, MN, USA). Quest™ Fluo-8 NW calcium assay kit was from AAT Bioquest (Sunnyvale, CA, USA).

### Mice

The investigation conformed to the Guide for the Care and Use of Laboratory Animals (Institute of Laboratory Animal Resources, eighth edition, 2011), and all animal experiments were performed in accordance with the approved guidelines by the Animal Care and Utilization Committee of the National Yang-Ming University (#1031269). B6.Cg-Tg(APPswe, PSEN1dE9)85Dbo/J (APP/PS1 Tg) mice and TRPA1^−/−^ mice were purchased from Jackson Laboratory (Bar Harbor, ME, USA) and were backcrossed to C57BL mice for at least 10 generations to ensure genetic homogeneity. For APP/PS1 Tg/TRPA1^−/−^ mice, TRPA1^−/−^ mice were crossed with the APP/PS1 Tg background, and the genotypes were confirmed by PCR of genomic DNA. Mice were housed in barrier facilities, maintained on a 12-h/12-h dark cycle. Temperature (22 °C) and humidity (40-60 %) of the vivarium were tightly controlled. Mice were group-housed 3–4 per cage and fed a regular chow diet, which contained 4.5 % fat by weight (0.02 % cholesterol) (Newco Distributors, Redwood, CA, USA). At the end of the experiment, mice were euthanized with CO_2_, then brains were harvested for histological analysis and stored at −80 °C. The isolated brains were homogenized and lysates were subjected to western blot analysis.

### Western blot analysis

Cells and brain tissues were lysed in immunoprecipitation lysis buffer (50 mmol/L Tris pH 7.5, 5 mmol/L EDTA, 300 mmol/L NaCl, 1 % Triton X-100, 1 mmol/L phenylmethylsulfonyl fluoride, 10 μg/mL leupeptin and 10 μg/mL aprotinin). Aliquots of brain lysates or cell lysates were separated on SDS-PAGE, transferred to membranes and immunoblotted with primary antibodies (1:1000), then horseradish peroxidase-conjugated secondary antibody (1:1000). Bands were revealed by use of an enzyme-linked chemiluminescence detection kit (PerkimElmer, Waltham, MA) and density was quantified by use of Imagequant 5.2 (Healthcare Bio-Sciences, Philadelphia, PA).

### Immunohistochemistry staining

The brain sections were fixed in 4 % paraformaldehyde and 15-μm cross sections were prepared. Sections were incubated with retrieval buffer for 10 min, blocked with 2 % BSA for 60 min and incubated with primary antibody (1:100) overnight at 4 °C, then FITC- or Texas red-conjugated secondary antibody (1:400) for 1 h at 37 °C. Antigenic sites were visualized under a Nikon TE2000-U microscope (Tokyo) with QCapture Pro 6.0 software (QImaging, BC, Canada).

### Fibrilization of Aβ_1–42_

Aβ_1–42_ was purchased from American Peptide Co. (Sunnyvale, CA, USA), solubilized in sterile water (1 mg/mL) and incubated 1 to 7 days at 37 °C for fibrillation as described [31–33]. The level of Aβ fibrilization on a 16.5 % tricine gel was examined by western blot analysis (Additional file 1: Figure S1).

### Cell culture

The primary culture of astrocytes and neurons was prepared as described [24, 34]. Briefly, the cortex and hippocampus were isolated from pups on postnatal 1 day and loosely homogenized by use of a sterile razor blade in DMEM/F12 (HyClone, Logan, UT). Tissues were digested with 0.01 % trypsin and incubated at 37 °C for 25 min; the cell suspension was titrated by use of a 70-μm nylon mesh. Isolated cells were seeded onto 75-mm flasks and incubated for 7 days in DMEM/F12 supplemented with 10 % FBS, 100 U/mL penicillin and 100 μg/mL streptomycin at 37 °C. Cells were re-suspended, followed by orbital shaking at 180 rpm for 24 h to remove microglia and oligodendrocytes. The purified astrocytes that tightly adhered to the bottom of the flasks were then detached with trypsin and seeded onto culture dishes and incubated for an additional 7 days to return to a resting state. The primary neuron culture was prepared as follows: the cortex was isolated from pups on postnatal 1 day and loosely homogenized by use of a sterile razor blade in DMEM (HyClone, Logan, UT). Isolated cells were seeded onto 3.5-cm dishes and incubated in Neurobasal media supplemented with 2 % B-27 supplement, 0.25 % GlutaMAX, 10 % FBS, 100 U/mL penicillin and 100 μg/mL streptomycin (Thermo Fisher Scientific, Waltham, MA, USA) at 37 °C. Cells were treated with cytosine-1-β-D-arabinofuranoside (10 μM) from day 2 and half the medium was changed every 3 days for 14 days’ culture. Human embryonic kidney 293 (HEK293) cells and mouse brain microvascular endothelial cells (BMECs), bEnd.3 cells, were cultured in DMEM supplemented with 10 % FBS, 100 U/mL penicillin and 100 μg/mL streptomycin. The growth media was replaced every other day.

### Plasmid construction and transient transfection

The coding region for the human TRPA1 DNA fragment was cloned into a pCMV5 N-Flag vector with MluI and HindIII restriction sites. The sequence of isolated DNA fragments was confirmed by sequence analysis. TurboFect was used for transient transfection experiments according to the manufacturer’s instructions. Briefly, 1 μg of vector or TRPA1 plasmid was transfected into HEK293 cells. Transfected cells were used in further experiments.

### Detection of Ca^2+^ influx

Primary astrocytes or HEK293 cells were pretreated with Fluo-8NW dye for 1 h, then medium was replaced with fresh medium containing test compounds. The intensity of fluorescence was evaluated by fluorometry (Molecular Devices, Sunnyvale, CA, USA) with 490-nm excitation and 525-nm emission. Images were captured under a TE2000-U fluorescence microscope and quantified with use of QCapture Pro 6.0.

### Measurement of PP2B activity

The activity of PP2B in primary astrocytes or fresh brain lysates was measured by use of a cellular PP2B activity kit.

### Measurement of inflammatory cytokines

The concentrations of inflammatory cytokines including IL-1β, IL-4, IL-6 and IL-10 in culture medium or brain lysates were measured by use of ELISA kits.

### Measurement of DNA-binding activity on NF-κB and NFAT

The DNA-binding activity of NF-κB and NFAT in primary astrocytes and brain was measured by use of ELISA kits.

### Immunocytochemical staining

Primary astrocytes were fixed with 4 % paraformaldehyde for 30 min, blocked with 2 % BSA for 30 min and incubated with primary antibodies (1:100), then FITC- or Texas red-conjugated secondary antibodies (1:400). Cellular images were viewed under a TE2000-U fluorescence microscope and quantified with use of QCapture Pro 6.0.

### Open field activity

The locomotor activity of mice was assessed in a cage (length × width × height: 28.5 × 28.5 × 30 cm). Mice were placed in the central of the cage and allowed to explore the open field for 5 min. The behavior was recorded by video, and the movement distance, percentage of resting time in the zone and trajectory were calculated for each mouse by use of Smart v3.0 software with the Panlab Harvard apparatus (Cornellà, Barcelona, Spain). The floor and internal walls were cleaned with ethanol between each trial.

### Nest-building test

The nest-building test was performed as described [35]. Each mouse was housed in single cages containing two pieces of cotton (5 × 5 cm). The presence and quality of nests built were recorded by the nesting score, measured on a 5-point scale: 1 = cotton not noticeably touched, 2 = cotton partially torn up, 3 = mostly shredded cotton but often no identifiable nest location, 4 = a markedly nesting site but flat nest, and 5 = a (near) perfect nest. Nesting score was recorded manually at 72-h intervals.

### Y-maze test

The Y-maze test was performed as described [36]. The Y-maze apparatus consists of three arms of channels made of stainless steel joined in the middle to form a “Y” shape. The mice were placed into one of the arms (start arm) and allowed to explore the maze with only one of the arms closed for 10 min (training trial). After 3 h, mice were placed back in the start arm of the Y maze. Then, mice were allowed to explore all three arms freely for 5 min (test trial). The number of entries into each arm, the distance of movement and the first choice of entry were assessed in video recordings.

### Morris water maze (MWM)

MWM was performed as described [36]. A large circular tank (0.8 m diameter, 0.4 m depth) was filled with water (25 ± 1 °C, 20 cm depth), and the escape platform (8 × 4 cm) was submerged 1 cm below the surface. The training section was monitored by a video system. The escape latency and trajectory of swimming were recorded for each mouse. The hidden platform was located at the center of one of the four quadrants in the tank. The location of the platform was fixed throughout the testing. Mice had to navigate using extra-maze cues that were placed on the walls of the maze. From days 1 to 4, mice went through three trials with an inter-trial interval of 5 min. The mouse was placed into the tank facing the side wall randomly at one of four start locations and allowed to swim until it found the platform or for a maximum of 120 sec. Mice that failed to find the platform within 120 sec were guided toward the platform. The animal then remained on the platform for 20 sec before being removed from the pool. The day after the hidden platform training, a probe trial was conducted to determine whether the mouse used a spatial strategy to find the platform. On day 5, the platform was removed from the pool and the mouse was allowed to swim freely for 120 sec. The proportion of time spent in each quadrant of the pool and the number of times the mouse crossed the former position of the hidden platform were recorded.

### Statistical analysis

Results are presented as mean ± SEM. Data from cell studies were evaluated by non-parametric tests. Mann-Whitney *U* test was used to compare 2 independent groups. Kruskal-Wallis followed by Bonferroni post-hoc analyses was used to account for multiple testing. Data from animal studies were evaluated by parametric tests. Two-way ANOVA followed by LSD test was used for multiple comparisons. The reagent effect and genotypic effect were two independent factors for this analysis. SPSS v20.0 (SPSS Inc, Chicago, IL) was used for analysis. Differences were considered statistically significant at *P* < 0.05.

## Results

### The expression of TRPA1 channels is upregulated in astrocytes of AD lesions

**Fig. 1:**
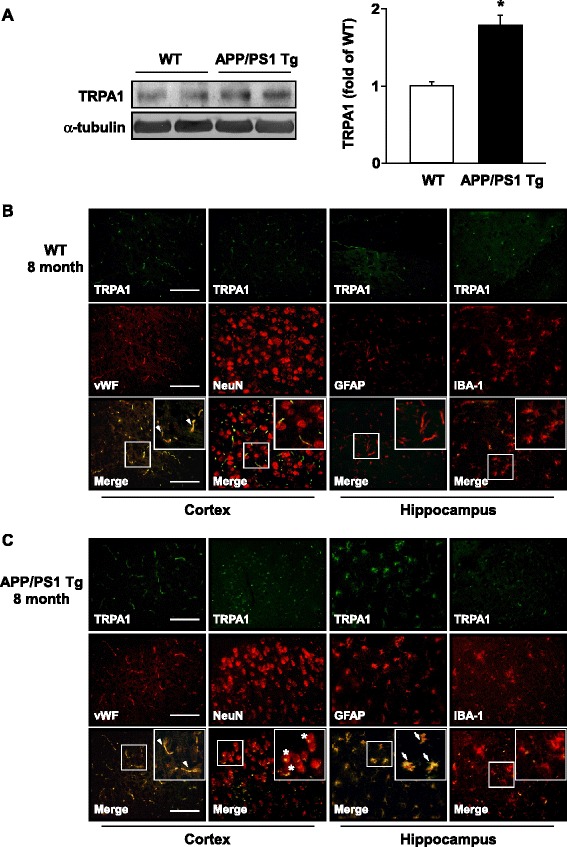
Expression and localization of TRPA1 channels in wild-type (WT) and APP/PS1 Tg mice. (**a**) Brains were harvested from WT and APP/PS1 Tg mice at 8 months old. Western blot analysis of protein levels of TRPA1 and α-tubulin. Data are mean ± SEM from 6 mice in each group. *, *P* < 0.05 vs. WT mice. (**b**, **c**) Immunohistochemistry of specimens of cortex and hippocampus from 8-month-old WT and APP/PS1 Tg mice with the antibodies anti-TRPA1, anti-vWF (endothelial cell marker), anti-NeuN (neuron marker), anti-GFAP (astrocyte marker) and anti-IBA-1 (microglia marker), then FITC- or Texas red-conjugated secondary antibody. Bar = 50 μm. vWF-positive cells denoted endothelial cells, NeuN-positive cells denoted neurons, and GFAP-positive cells denoted astrocytes, as indicated by arrowheads, stars or arrows, respectively

To explore the possible participation of TRPA1 channels in the pathogenesis of AD, we investigated the expression and distribution of TRPA1 channels in brains from WT and APP/PS1 Tg mice. The protein level of TRPA1 channels was higher in APP/PS1 Tg than WT mice at 8 months old (Fig. 1a, *n* = 6, *P* < 0.05). However, the protein level did not differ between APP/PS1 Tg and WT mice at 3 months old (Additional file 1: Figure S2, *n* = 6, *P* > 0.05). In the cortex, TRPA1 channels were expressed in endothelial cells of both WT and APP/PS1 Tg mice (Fig. 1b, c). However, the immunoreactivity of TRPA1 channels in neurons was observed only in APP/PS1 Tg mice (Fig. 1b, c). In the hippocampus, TRPA1 channels were expressed in astrocytes of both WT and APP/PS1 Tg mice; however, TRPA1 level in hippocampus astrocytes was greater in APP/PS1 Tg than WT mice (Fig. 1b, c). In WT mice, the protein level of TRPA1 was much lower in neurons than astrocytes and BMECs (Additional file 1: Figure S3). TRPA1 may play an important role in astrocyte biology in AD pathogenesis.

### Genetic loss of function of TRPA1 channels on an APP/PS1 Tg background improves nest building and spatial learning and memory

**Fig. 2:**
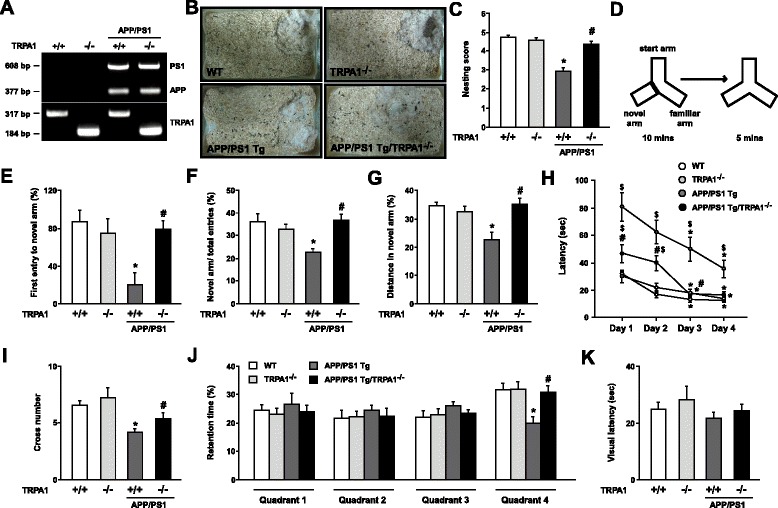
Loss of function of TRPA1 channels improves nest building and spatial learning and memory in APP/PS1 Tg mice. (**a**) PCR genotyping of representative WT, TRPA1^−/−^, APP/PS1 Tg and APP/PS1 Tg/TRPA1^−/−^ mice. PS1 = 608 bp, APP = 377 bp, TRPA1^+/+^ = 317 bp and TRPA1^−/−^ = 184 bp. (**b**, **c**) Representative examples and the score for nest building for 8-month-old WT, TRPA1^−/−^, APP/PS1 Tg and APP/PS1 Tg/TRPA1^−/−^ mice. (**d-g**) Schematic diagram of Y-maze experimental design and ratio of first entry, number of entries and distance moved in the novel arm. (**h**) Morris water maze (MWM) test of learning patterns verified on days 1 to 4. (**i**, **j**) At day 5 after training, the number of times crossing the hind platform and retention times in the all quadrants. (**k**) The latency of arrival to the visual platform. Data are mean ± SEM from 8 mice in each group. *, *P* < 0.05 vs. WT mice or day 1 (h panel), #, *P* < 0.05 vs. APP/PS1 Tg mice, $ *P* < 0.05 vs. WT mice (h panel)

To assess the effect of TRPA1 channels in the pathophysiology of AD, we generated APP/PS1 Tg/TRPA1^−/−^ mice (Fig. 2a). APP/PS1 Tg/TRPA1^−/−^ mice showed better performance in nest building than APP/PS1 Tg mice at 8 months old (Fig. 2b, c, *n* = 8, *P* < 0.05). In the Y-maze test, 8-month-old APP/PS1 Tg/TRPA1^−/−^ mice showed increased preference for first entry to a novel arm; the ratio of entry number and moving distance in the novel arm was greater in these mice than in 8-month-old APP/PS1 Tg mice (Fig. 2d-g, *n* = 8, *P* < 0.05). Next, the MWM test was performed to evaluate the ability of spatial learning and memory of mice. With the hidden platform test, both APP/PS1 Tg and APP/PS1 Tg/TRPA1^−/−^ mice learned the location of the hidden platform on day 3 and 4; however, 8-month-old APP/PS1 Tg/TRPA1^−/−^ mice showed shorter escape latencies, which indicates better learning patterns, than APP/PS1 Tg mice with a 4-day training (Fig. 2h, *n* = 8, *P* < 0.05). Moreover, APP/PS1 Tg/TRPA1^−/−^ mice showed increased number of times crossing the hidden platform and retention times in the target quadrant (quadrant 4) at day 5 after training (Fig. 2i, j, *n* = 8, *P* < 0.05). The genotypes did not differ in swimming to the visual platform test (Fig. 2k). However, WT, TRPA1^−/−^, APP/PS1 Tg and APP/PS1 Tg/TRPA1^−/−^ mice showed no difference in the above behaviors and locomotion (Additional file 1: Figure S4, *n* = 8, *P* > 0.05). These findings suggest that the TRPA1 channel might be a key regulator in AD-related cognitive performance behavior and spatial learning and memory.

### Genetic ablation of function of TRPA1 channels on an APP/PS1 Tg background decreases Aβ deposition in brain lesions and mitigates inflammation but aggravates astrogliosis

**Fig. 3:**
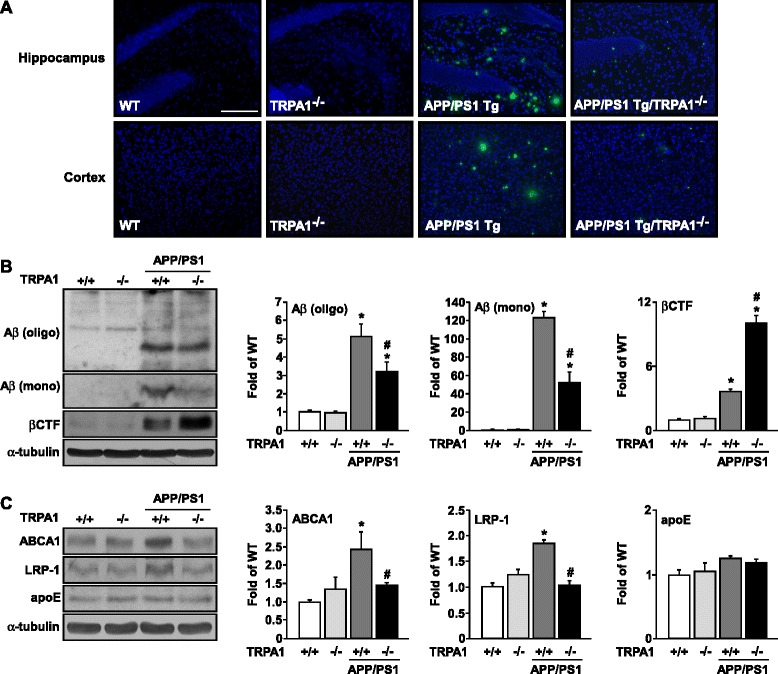
Ablation of TRPA1 channel function decreases Aβ deposition in brain lesions of APP/PS1 Tg mice. (**a**) Immunostaining of specimens of hippocampus and cortex from 8-month-old WT, TRPA1^−/−^, APP/PS1 Tg and APP/PS1 Tg/TRPA1^−/−^ mice with anti-Aβ antibody, then FITC-conjugated secondary antibody. (**b**, **c**) Western blot analysis of protein levels of oligomer (oligo) or monomer (mono) Aβ, βCTF, ABCA1, LRP-1, apoE and α-tubulin in brain specimens. Bar = 100 μm. Data are mean ± SEM from 8 mice in each group. *, *P* < 0.05 vs. WT mice. #, *P* < 0.05 vs. APP/PS1 Tg mice

**Fig. 4:**
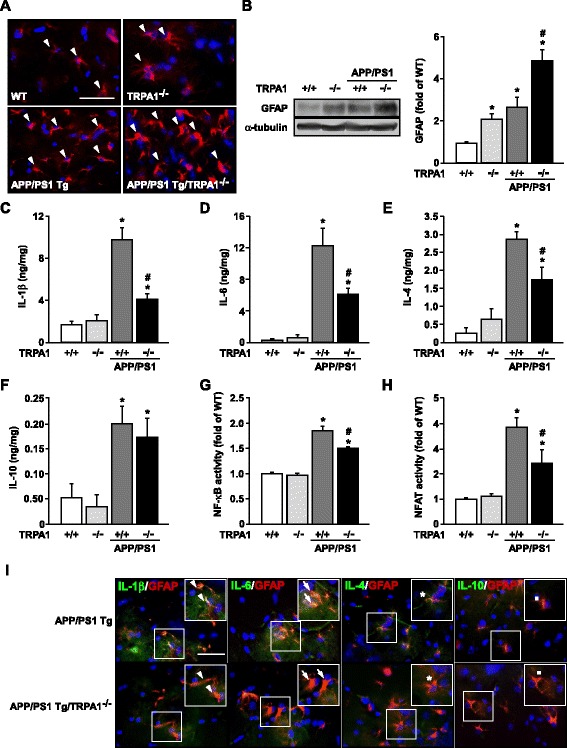
Genetic disruption of TRPA1 channel function increases astrogliosis but decreases inflammation in hippocampus of APP/PS1 Tg mice. (**a**) Immunostaining of hippocampus specimens from 8-month-old WT, TRPA1^−/−^, APP/PS1 Tg and APP/PS1 Tg/TRPA1^−/−^ mice with anti-GFAP antibody, then Texas red-conjugated secondary antibody. (**b**) Western blot analysis of protein levels of GFAP and α-tubulin. ELISA of (**c-f**) IL-1β, IL-6, IL-4 and IL-10 secretion and (**g**, **h**) NF-κB and NFAT DNA binding activity in brain specimens from 8-month-old WT, TRPA1^−/−^, APP/PS1 Tg and APP/PS1 Tg/TRPA1^−/−^ mice. (**i**) Immunostaining of hippocampus specimens from 8-month-old APP/PS1 Tg and APP/PS1 Tg/TRPA1^−/−^ mice with the antibodies anti-GFAP, anti-IL-1β, IL-6, IL-4 or IL-10, then FITC- or Texas red-conjugated secondary antibody. Bar = 50 μm. GFAP-positive cells (red color) denote astrocytes; IL-1β-, IL-6-, IL-4- and IL-10-positive astrocytes (green color) are indicated by arrowheads, arrows, stars or dots, respectively. Data are mean ± SEM from 8 mice in each group. *, *P* < 0.05 vs. WT mice. #, *P* < 0.05 vs. APP/PS1 Tg mice

To investigate the potential molecular mechanism of TRPA1 channels underlying AD pathogenesis and Aβ-triggered inflammation, we examined Aβ plaque deposition in AD lesions of APP/PS1 Tg and APP/PS1 Tg/TRPA1^−/−^ mice. Aβ plaque deposition was markedly decreased in cortex and hippocampus of APP/PS1 Tg/TRPA1^−/−^ mice (Fig. 3a). Western blot results of Aβ in both oligomers and monomers and βCTF within brain lysates from APP/PS1 Tg and APP/PS1 Tg/TRPA1^−/−^ mice supported the decreased Aβ plaque deposition in APP/PS1 Tg/TRPA1^−/−^ mice (Fig. 3b, *n* = 8, *P* < 0.05). The expression of ABCA1, LRP-1 (*n* = 8, *P* < 0.05) but not apoE (*n* = 8, *P* > 0.05) was decreased in APP/PS1 Tg/TRPA1^−/−^ mice (Fig. 3c). However, astrogliosis was more severe in APP/PS1 Tg/TRPA1^−/−^ than APP/PS1 Tg mice (Fig. 4a, b, *n* = 8, *P* < 0.05). Functional loss of TRPA1 channels markedly reduced the levels of the inflammatory cytokines IL-1β, IL-6 and IL-4 (*n* = 8, *P* < 0.05) but not anti-inflammatory cytokine IL-10 (*n* = 8, *P* > 0.05) in AD brains (Fig. 4c-f). We next evaluated the activity of NF-κB and NFAT, major transcription factors regulating the expression of IL-1β, IL-6, IL-4 and IL-10 [37–40]. DNA-binding activities of both NF-κB and NFAT were lower with functional deletion of the TRPA1 channel in APP/PS1 Tg/TRPA1^−/−^ mice than in APP/PS1 Tg mice (Fig. 4g, h, *n* = 8, *P* < 0.05). Immunohistochemistry revealed that IL-1β, IL-6, IL-4 and IL-10 were mainly expressed in astrocytes of APP/PS1 Tg and APP/PS1 Tg/TRPA1^−/−^ mice (Fig. 4i). However, functional ablation of TRPA1 channels on the C57BL background did not induce Aβ deposition and inflammation in brains (Fig. 3, 4, *n* = 8, *P* > 0.05) but increased astrogliosis (Fig. 4a, b, *n* = 8, *P* < 0.05). The TRPA1 channel may be a key player in regulating Aβ metabolism and inflammatory responses during AD development.

### Aβ − elicited increase in intracellular Ca^2+^ in astrocytes is TRPA1-dependent

**Fig. 5:**
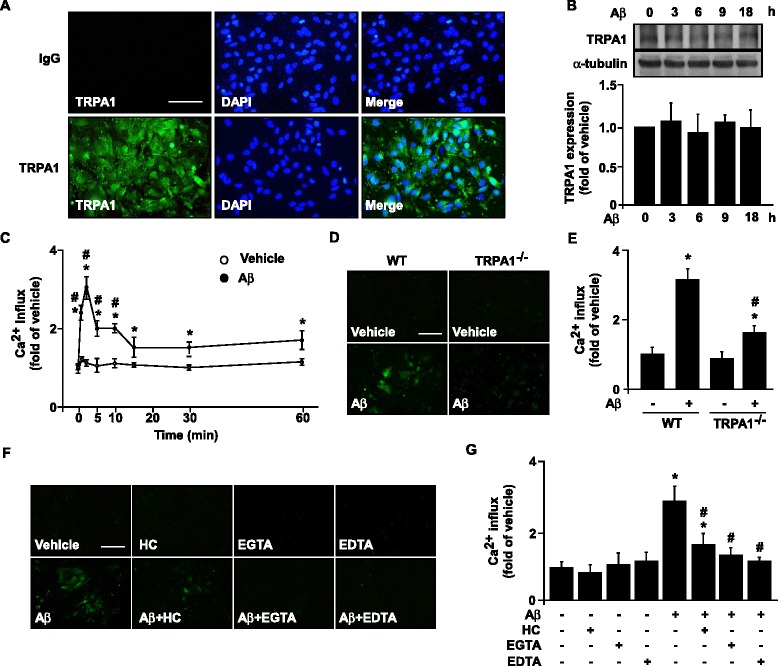
Aβ elicits TRPA1-dependent Ca^2+^ influx in astrocytes. (**a**) Immunostaining of primary astrocytes from WT mice with anti-IgG and anti-TRPA1 antibody, then FITC-conjugated secondary antibody. (**b**) Western blot analysis of TRPA1 and α-tubulin protein levels in astrocytes treated with 2 μM Aβ for the indicated times (0–18 h). (**c**) Ca^2+^ influx in astrocytes treated with 2 μM Aβ for 0–60 min. Fluo-8 calcium assay of intracellular level of Ca^2+^ in (**d**, **e**) primary astrocytes from WT and TRPA1^−/−^ mice treated with Aβ (2 μM) for 2 min and (**f**, **g**) astrocytes pretreated with HC030031 (HC) 10 μM, EGTA 0.5 mM and EDTA 0.5 mM for 2 h, then treated with Aβ for 2 min. Fluorescence images were photographed by fluorescence microscopy. Bar = 100 μm. Data are mean ± SEM from 5 independent experiments. *, *P* < 0.05 vs. vehicle or 0 min. #, *P* < 0.05 vs. WT Aβ treatment or vehicle

**Fig. 6:**
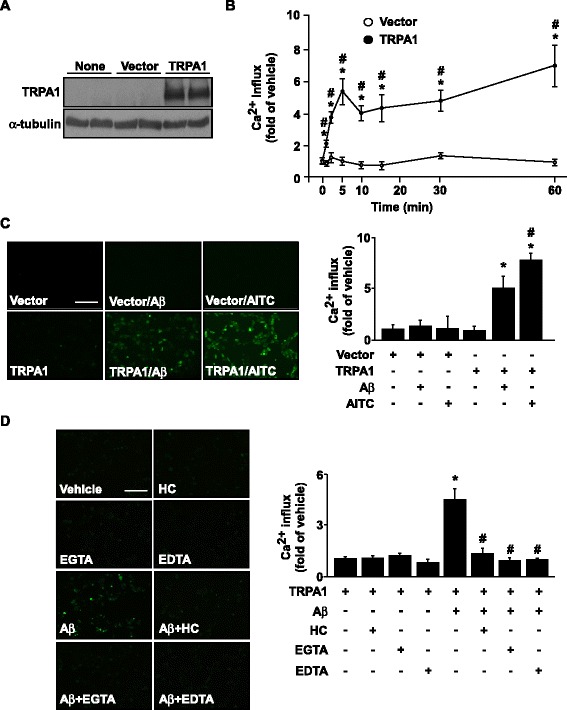
The essential role of TRPA1 in Aβ-induced calcium influx in HEK293 cells. (**a**) Western blot analysis of TRPA1 and α-tubulin protein levels in non-treated, vector- or TRPA1-transfected HEK293 cells. (**b**) Ca^2+^ influx in HEK293 cells transfected with vector or TRPA1 plasmid, then treated with Aβ (2 μM) for 0–60 min. Fluo-8 calcium assay of the intracellular level of Ca^2+^ in (**c**) HEK293 cells transfected with vector or TRPA1 plasmid for 24 h, then treated with Aβ (2 μM) or AITC (10 μM, a TRPA1 agonist) for 5 min and (**d**) after transfection with TRPA1 plasmid, HEK293 cells pretreated with a TRPA1 antagonist HC030031 (HC) 10 μM, EGTA 0.5 mM and EDTA 0.5 mM for 2 h, then with Aβ for 5 min. Fluorescence images were photographed by fluorescence microscopy. Bar = 100 μm. Data are mean ± SEM from 5 independent experiments. *, *P* < 0.05 vs. 0 min or TRPA1 transfection alone. #, *P* < 0.05 vs. vector-transfection alone or TRPA1 transfection + Aβ treatment

To examine the potential role of TRPA1 channels in the detrimental effect of Aβ on AD, we first detected the expression of the TRPA1 channel in astrocytes (Fig. 5a). Treatment with fibrilized Aβ did not changes the expression of TRPA1 channels in astrocytes over time (Fig. 5b, *n* = 5, *P* > 0.05). We then investigated whether Aβ could induce functional activation of TRPA1 in astrocytes by treating WT astrocytes with 2 μM Aβ for the indicated times. Compared with vehicle treatment, Aβ treatment time-dependently promoted Ca^2+^ influx within 60 min. The elevated intracellular Ca^2+^ level occurred as early as 2 min after treatment (Fig. 5c, *n* = 5, *P *< 0.05). Additionally, functional deletion of TRPA1, treatment with TRPA1 pharmacological inhibitor HC030031, and pretreatment with EGTA or EDTA all abrogated the fibrilized Aβ-increased intracellular Ca^2+^ level in astrocytes (Fig. 5d-g, *n* = 5, *P* < 0.05). To further investigate the involvement of TRPA1 in Aβ-induced Ca^2+^ influx, we re-expressed full-length human TRPA1 channels in HEK293 cells, which lack TRPA1 channels. TRPA1 channels were successfully expressed in HEK293 cells (Fig. 6a). Treatment with both Aβ and AITC, a TRPA1 selective agonist, significantly increased intracellular Ca^2+^ level in TRPA1-positive HEK293 cells as compared with empty vector–transfected cells, with the increase in intracellular Ca^2+^ level observed with 1- to 60-min Aβ treatment (Fig. 6b, c, *n* = 5, *P* < 0.05). Blockage of TRPA1 activation by the TRPA1 selective antagonist HC030031 or removal of extracellular Ca^2+^ by EGTA or EDTA abrogated Aβ-induced Ca^2+^ influx in TRPA1-reexpressed HEK293 cells (Fig. 6d, *n* = 5, *P* < 0.05). Therefore, TRPA1 is essential for Aβ-induced Ca^2+^ mobilization.

### TRPA1 − Ca^2+^ signaling plays a crucial role in Aβ-mediated PP2B activation, inflammatory responses and astrogliosis in astrocytes

**Fig. 7:**
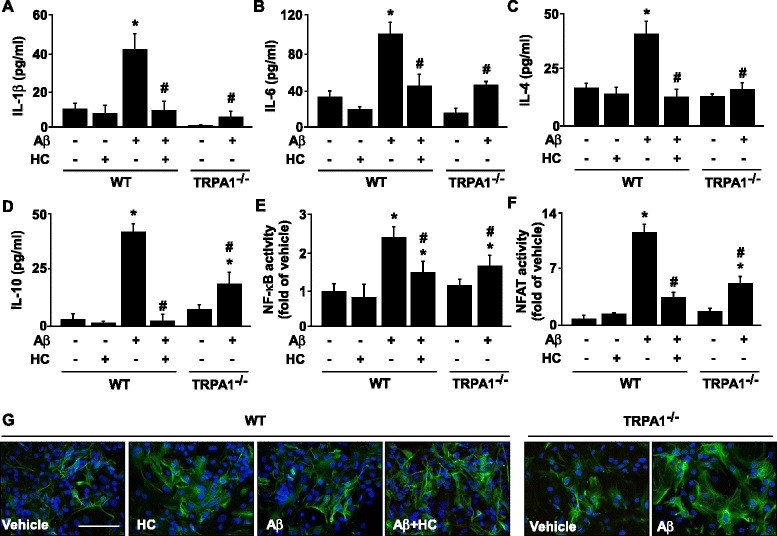
TRPA1 is crucial in regulating Aβ-induced inflammatory response and astrogliosis in astrocytes. Primary astrocytes from WT and TRPA1^−/−^ mice were pretreated with HC030031 (HC; 10 μM) for 2 h, then with Aβ (2 μM) for 24 h (**a-d**) or 1 h (**e**, **f**). ELISA of (**a-d**) IL-1β, IL-6, IL-4 and IL-10 secretion in culture medium and (**e**, **f**) NF-κB and NFAT DNA binding activity in cellular lysates. (**g**) Immunostaining of astrocytes from treated WT and TRPA1^−/−^ mice with anti-GFAP antibody, then FITC-conjugated secondary antibody. Bar = 100 μm. Data are mean ± SEM from 5 independent experiments. *, *P* < 0.05 vs. vehicle. #, *P* < 0.05 vs. WT Aβ treatment

**Fig. 8:**
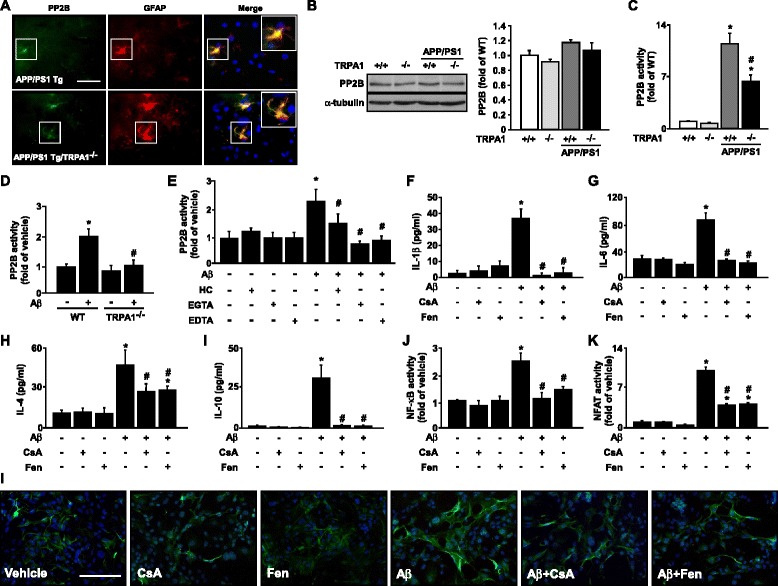
PP2B activation is vital in Aβ-induced inflammation in astrocytes. (**a**) Immunostaining of hippocampus specimens from 8-month-old APP/PS1 Tg and APP/PS1 Tg/TRPA1^−/−^ mice with anti-GFAP and anti-PP2B antibodies, then FITC- or Texas red-conjugated secondary antibody. Bar = 50 μm. GFAP-positive cells (red color) denote astrocytes and FITC-positive cells (green color) denote PP2B protein. (**b** and **c**) Western blot analysis of PP2B and its activity in brain lysates from 8-month-old WT, TRPA1^−/−^, APP/PS1 Tg and APP/PS1 Tg/TRPA1^−/−^ mice. (**d**) Primary astrocytes from WT and TRPA1^−/−^ mice were treated with Aβ (2 μM) for 30 min. (**e**) WT astrocytes were pretreated with HC (10 μM), 0.5 mM EGTA or 0.5 mM EDTA for 2 h, then Aβ (2 μM) for 30 min. (**f**-**k**) WT astrocytes were pretreated with cyclosporin A (CsA, 100 nM) or fenvalerate (Fen, 100 nM) for 2 h, then with Aβ (2 μM) for 24 h (**f-i**) or 1 h (**j** and **k**). ELISA of (**f-i**) IL-1β, IL-6, IL-4 and IL-10 in culture medium and (**j**, **k**) NF-κB and NFAT DNA binding activity in cell lysates. (**l**) Immunostaining of astrocytes from treated WT mice with anti-GFAP antibody, then FITC-conjugated secondary antibody. Bar = 100 μm. Data are mean ± SEM from 5 independent *in vitro* experiments or from 8 mice in each group. *, *P* < 0.05 vs. vehicle or WT mice. #, *P* < 0.05 vs. Aβ treatment or APP/PS1 Tg mice

**Fig. 9:**
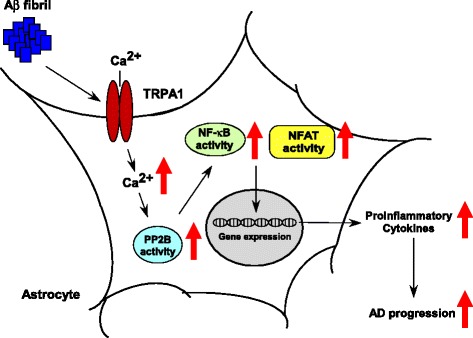
TRPA1 − Ca^2+^ − PP2B signaling pathway in Aβ − triggered activation of NF-κB and NFAT and inflammation in astrocytes. A proposed mechanism by which TRPA1 channel regulates Aβ-mediated inflammatory response in astrocytes. Aβ fibril activates PP2B, NF-κB and NFAT and, ultimately, production of pro-inflammatory cytokines in astrocytes

Emerging evidence indicates that Aβ-elevated intracellular Ca^2+^ level may trigger pro-inflammatory cytokine production by activating PP2B and its downstream transcription factors such as NF-κB and NFAT [14–16]. Therefore, we examined whether TRPA1-Ca^2+^ signaling is involved in Aβ-induced activation of PP2B, the DNA-binding activities of its downstream targets NF-κB and NFAT, and subsequent inflammatory responses both *in vivo* and *in vitro*. We investigated the significance of TRPA1 channels in astrocytes with Aβ treatment in terms of inflammatory responses and astrogliosis. Low fibrilization Aβ triggered the production of IL-4 and IL-10 in astrocytes, whereas high fibrilization stimulated the production of IL-1β and IL-6 (Additional file 1: Figure S1, *n* = 5, *P* < 0.05). With high fibrillation of Aβ, functional inhibition of TRPA1 channel activity by HC030031 or genetic manipulation abrogated the Aβ-mediated production of IL-1β, IL-6, IL-4 and IL-10 in astrocytes (Fig. 7a-d, *n* = 5, *P* < 0.05). Moreover, pretreatment with HC030031 or genetic disruption of TRPA1 function diminished the Aβ-induced activation of NF-κB and NFAT (Fig. 7e, f, *n* = 5, *P* < 0.05). Exposure of astrocytes to fibrilized Aβ elicited astrogliosis, which was aggravated by pharmacological inhibition or genetic disruption of TRPA1 channel activity (Fig. 7g). As well, PP2B protein was expressed in hippocampus astrocytes of both APP/PS1 Tg and APP/PS1 Tg/TRPA1^−/−^ mice (Fig. 8a). Functional ablation of TRPA1 channels in mice on a C57BL or APP/PS1 Tg background did not affect the protein expression of PP2B (Fig. 8b). However, genetic deletion of TRPA1 in mice on an APP/PS1 Tg background decreased PP2B activity in AD brains (Fig. 8c, n = 8, P < 0.05). Genetic ablation of TRPA1 channel function, treatment with HC030031, or removal of Ca^2+^ by EGTA or EDTA all diminished the Aβ-increased PP2B activity in astrocytes (Fig. 8d, e, *n* = 5, *P* < 0.05). Moreover, pharmacological inhibition of PP2B activity by CsA or Fen prevented the Aβ-induced production of IL-1β, IL-6, IL-4 and IL-10 (Fig. 8f-i, *n* = 5, *P* < 0.05) and activation of NF-κB and NFAT in astrocytes (Fig. 8j, k, *n* = 5, *P* < 0.05), as well as astrogliosis (Fig. 8l). Collectively, these findings strongly suggest that activation of a TRPA1 − Ca^2+^ − PP2B signaling cascade might be an important event in Aβ-induced inflammation of astrocytes and AD pathogenesis (Fig. 9).

## Discussion

Our study demonstrated the novel role of the TRPA1 channel in astrocytes under an Aβ-elicited inflammatory environment and thus its potential involvement in AD pathogenesis. The protein level of the TRPA1 channel was increased in astrocytes of APP/PS1 Tg mice at 8 months old as compared with WT mice at the same age. As well, loss of function of the TRPA1 channel impeded AD progression, as evidenced by improved nest building ability, spatial learning and memory and decreased Aβ plaque deposition and cytokine production in APP/PS1 Tg mice. *In vitro*, TRPA1 channels mediated the Aβ-triggered Ca^2+^ influx and inflammation in astrocytes. Gain of function of TRPA1 in TRP channel–deficient HEK293 cells further supports that TRPA1 activation is crucial in Aβ-mediated Ca^2+^ influx. Aβ evoked TRPA1-Ca^2+^ signaling, which in turn activated PP2B, NF-κB and NFAT. The activation of these proteins increased the production of inflammatory cytokines. This inflammatory cascade in our model agreed with findings by Fernandez et al. and Furman et al., who established that increased Ca^2+^ influx is a key event for activation of PP2B signaling and inflammation in astrocytes [14–16]. Finally, behavioral analysis demonstrated that functional loss of TRPA1 ameliorated AD progression and improved neuropsychiatric signs, positively affecting cognition and spatial learning and memory. Collectively, our results suggest that the TRPA1 channel is involved in Aβ-triggered inflammatory responses of astrocytes and the development of AD.

The association of deregulated cellular Ca^2+^ homeostasis and AD pathogenesis has been established *in vitro* and *in vivo* [41–43]. Emerging evidence further supported the causal relationship between the impaired Ca^2+^ homeostasis and synaptic dysfunction and neuronal degeneration of AD [1]. Specifically, exposure of Aβ to neurons disrupts Ca^2+^ homeostasis and causes oxidative damage, thereby leading to neuronal death and synaptic dysfunction via multiple receptor-dependent pathways including NMDA receptor or voltage-gated Ca^2+^ channels [1]. However, research into Ca^2+^ homeostasis of astrocytes and its role in AD pathogenesis is still in its infancy. Several lines of evidence indicated that TRP channels such as canonical (TRPC), melastatin (TRPM), and vanilloid (TRPV) channels also play a role in AD pathogenesis [44, 45]. Especially, astrocyte-related inflammation is a key factor in AD progression [8, 9, 46, 47]. Aβ-induced Ca^2+^ influx is closely associated with the inflammatory responses in astrocytes [11–13]. In astrocytes, Aβ elicits the production of ROS and nitric oxide (NO), which can activate TRPM2, TRPM7, TRPC5 and TRPV1 and increase the intracellular level of Ca^2+^, thereby leading to AD-related events in the brain including neurodegeneration and inflammation [30].

However, how TRPA1 activation, particularly in astrocytes, contributes to AD pathogenesis has not been investigated. Our results demonstrated that Aβ triggered TRPA1-dependent Ca^2+^ influx and subsequently increased PP2B activity, which promoted activation of NF-κB and NFAT, thereby leading to production of pro-inflammatory cytokines from astrocytes. Most importantly, we observed these key events in our *in vivo* model. Of note, in astrocytes, both pretreatment with HC030031 and genetic loss of function of TRPA1 channels could only partially obstruct the Aβ-elicited Ca^2+^ influx. In contrast, pretreatment with EGTA and EDTA completely abolished Ca^2+^ influx induced by Aβ. These results imply the existence of other routes of Ca^2+^ influx triggered by Aβ. Indeed, several proteins regulating Ca^2+^ flow on Aβ challenge in astrocytes have been identified [42, 43]. In response to ligands, TRP channels can form homo- or heterotetramers or activate other TRP channels, thereby increasing intracellular Ca^2+^ level and activating downstream Ca^2+^-mediated signaling cascades [48, 49]. However, whether other TRP channels participate in the Aβ-induced TRPA1 channel activation and if so, how they activate the TRPA1 channel remains unknown.

PP2B activity of astrocytes is highly associated with neurodegenerative diseases [50, 51]. Increased numbers of PP2B-positive astrocytes were found in the immediate vicinity of extracellular Aβ deposition in patients with dementia and in an AD mouse model [50, 51]. In addition, disruption of Ca^2+^ homeostasis causes hyperactivity of PP2B signaling cascades, which can amplify the offset effect of Ca^2+^ dysregulation [15]. This notion was further supported by our findings that TRPA1-derived Ca^2+^ mobilization was critical in Aβ-activated PP2B, as evidenced by reduced PP2B activity in TRPA1 antagonist–treated or TRPA1-deficient astrocytes or brain tissues of APP/PS1 Tg/TRPA1^−/−^ mice. PP2B can lessen Akt activity its downstream signaling pathways, which are involved in inflammation and AD [52–54]. In fact, deletion of TRPA1 decreased PP2B activity but increased the phosphorylation of Akt in brain tissues of APP/PS1 Tg mice (Additional file 1: Figure S5). Thus, TRPA1 − Ca^2+^ − PP2B signaling may play an important role in AD progression. However, the detailed molecular mechanism by which PP2B regulates AD progression needs further investigation.

In the AD brain, Aβ plaques surrounding astrocytes can secrete inflammatory mediators to regulate neuroinflammation [55]. IL-1β, IL-4, and IL-6 are involved in the initiation and progression of AD by deregulating Aβ-mediated inflammation and APP metabolism [55–57]. In contrast, IL-10 can limit inflammation by reducing pro-inflammatory cytokines during AD pathogenesis [55]. In line with these findings, our data demonstrated lower levels of IL-1β, IL-4, IL-6, but IL-10 in APP/PS1 Tg/TRPA1^−/−^ mice than APP/PS1 Tg mice. Moreover, functional inhibition of TRPA1 abrogated the Aβ-triggered production of IL-1β, IL-4 and IL-6 in astrocytes. The transcriptional factors NF-κB and NFAT are key regulators in gene expression of IL-1β, IL-4 and IL-6 [37–40]. Consistently, we found that functional deletion of TRPA1 decreased Aβ-induced NF-κB and NFAT activity in astrocytes and mice. Nevertheless, the mechanism underlying TRPA1-mediated regulation of inflammation and AD pathogenesis needs further investigation.

Astrogliosis (referred to as reactive astrocytes) occurs prominently in response to central nervous system injury or remodeling [9, 58]. Although the biological function of astrogliosis is not fully understood, reactive astrocytes are intimately associated with Aβ plaque and involved in the regulation of neural protection and repair, glial scarring and neuro-inflammation in the pathogenesis of AD [58]. In addition, reactive astrocytes promote the clearance and degradation of Aβ in AD brain and thus limit the growth of Aβ plaque [59]. Interestingly, we found greater astrogliosis after treatment with a TRPA1 antagonist or functional deletion of TRPA1 *in vitro* and *in vivo*. In parallel, the neuro-inflammation was ameliorated with both pharmacological inhibition and genetic deletion of TRPA1 in Aβ-treated astrocytes and mouse brains. Activation of TRPA1-Ca^2+^ signaling might be critical in regulating Aβ-mediated astrogliosis and neuro-inflammation.

APP can be rapidly metabolized by post-translational proteolysis via an amyloidogenic or non-amyloidogenic pathway in neurons, and its role in AD is well established [3, 60]. In the amyloidogenic pathway, APP is cleaved by β-secretase, and N-terminal APP fragments and βCTF are produced and released. βCTF is then cleaved by γ-secretase and generates an amyloid precursor protein intracellular domain and toxic Aβ peptide (37–49 amino acids), which triggers inflammation and neuron dysfunction [3, 5, 60]. In contrast, in the non-amyloidogenic pathway, APP is cleaved by α-secretase and yields a soluble N-terminal APPα fragment and α-C-terminal fragment (αCTF). αCTF is then cleaved by γ-secretase to generate non-toxic p83 peptide (23–25 amino acids) [60, 61]. Disruption of Ca^2+^ homeostasis may deregulate APP processing and increase Aβ generation [61]. This notion was supported by our findings that ablation of TRPA1-Ca^2+^ signaling decreased Aβ production and accumulation in the AD mouse brain. Notably, our results further demonstrated that functional loss of TRPA1 channels increased the levels of βCTF but decreased that of Aβ in the AD mouse brain. Thus, TRPA1-Ca^2+^ signaling might regulate APP processing, especially at the step of cleavage of βCTF to Aβ generation.

Previous evidence linked cholesterol metabolism of the brain to Aβ clearance [62, 63]. Astrocyte-derived apoE-containing high-density lipoprotein-like particles play a central role in neural repair during AD development [62–64]. These particles can be transported by ABCA1 into the extracellular space to bind with Aβ and then are taken up by neurons, astrocytes and microglia through LRP-1 to remove the Aβ deposition [63]. The expression of ABCA1 and LRP-1 is increased with AD progression [65, 66]. We found that functional loss of TRPA1 channels decreased the expression of ABCA1 and LRP-1 without altering apoE expression and its co-localization with Aβ plaque in APP/PS1 Tg mice. As we described previously, Aβ accumulation was lower in the brain of APP/PS1 Tg/TRPA1^−/−^ than APP/PS1 Tg mice, so APP/PS1 Tg/TRPA1^−/−^ mice might not need such levels of ABCA1 and LRP-1 to clear Aβ. Nevertheless, we cannot conclude that TRPA1 channels are a negative regulator of Aβ clearance based on these observations.

## Conclusions

In conclusion, we provide experimental evidence to support the critical role of TRPA1-Ca^2+^ signaling in regulating Aβ-triggered inflammation and AD progression. The molecular mechanisms we established may provide important information for depicting the pathogenesis of AD and aid in the development of therapeutic interventions for AD and other neurodegenerative diseases.

## Additional file

Additional file 1: Supplementary figures. (PDF 547 kb)

## Abbreviations

ABCA1: ATP-binding cassette transporter A1
AD: Alzheimer’s disease
AITC: allyl isothiocyanate
apoE: apolipoprotein E
APP: amyloid precursor protein
Aβ: amyloid-β
αCTF: α-C-terminal fragment
BACE1: β-site APP cleavage enzyme 1
BSA: bovine serum albumin
βCTF: β-APP C-terminal fragment
Ca^2+^: calcium
EDTA: ethylenediaminetetraacetic acid
EGTA: ethylene glycol tetraacetic acid
GFAP: glial fibrillary acidic protein
HEK293: human embryonic kidney 293
IBA-1: ionized calcium-binding adapter molecule 1
IL: interleukin
LRP-1: LDLR-related protein 1
MWM: Morris water maze
NFAT: nuclear factor of activated T cells
NO: nitric oxide
PP2B: protein phosphatase 2B
PS1: presenilin 1
ROS: reactive oxygen species
TRPA1: transient receptor potential ankyrin 1
TRPC: transient receptor potential canonical
TRPM: transient receptor potential melastatin
TRPV: transient receptor potential vanilloid
vWF: von Willebrand factor
