# Supplementary material for: Role of transient receptor potential ankyrin 1 channels in Alzheimer’s disease
Source: J Neuroinflammation. 2016 Apr 27;13:92. doi: 10.1186/s12974-016-0557-z (PMC4847235; doi:10.1186/s12974-016-0557-z)
Supplement: Additional file 1: — Supplementary figures. (PDF 547 kb) [file 12974_2016_557_MOESM1_ESM.pdf]

Supplementary Information

Role of transient receptor potential ankyrin 1 channels in Alzheimer's disease

Kuan-I Lee, Hsueh-Te Lee, Hui-Ching Lin, Huey-Jen Tsay, Feng-Chuan Tsai,

Song-Kun Shyue, Tzong-Shyuan Lee

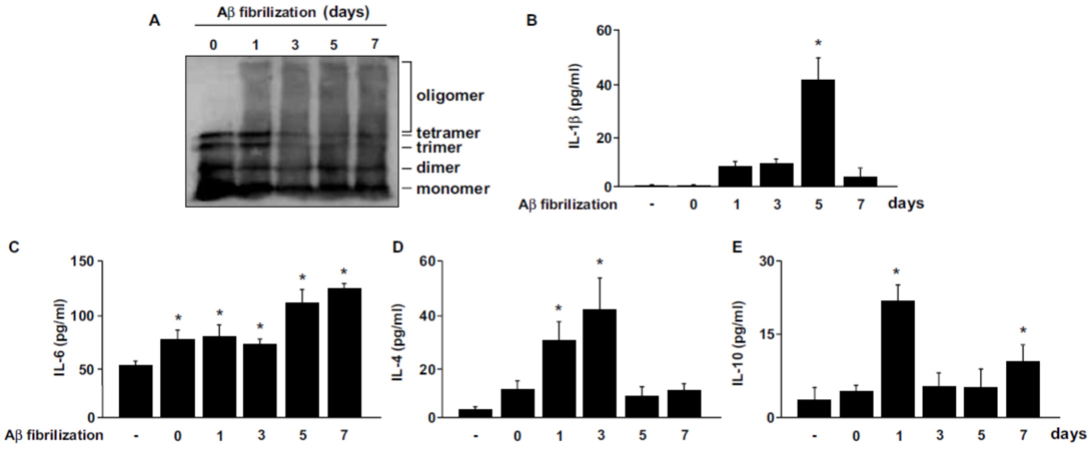

Supplementary Fig. S1. Different degree of Aβ fibrilization induces diverse inflammatory cytokine production in primary astrocytes. Aβ fibrilization was performed by incubating Aβ with sterile water at 37°C for 1-7 days. After 5 days of incubation, Aβ formed a robust high-molecular-weight oligomer. (**a**) Western blot analysis of Aβ protein level. (**b**-**e**) Primary astrocytes were treated with different fibrilization conditions of Aβ (2 μM) for 24 h. ELISA of IL-1β, IL-6, IL-4 and IL-10 secretion in culture medium of WT astrocytes. Data are mean ± SEM from 5 independent experiments. *, $P < 0.05$ vs. vehicle.

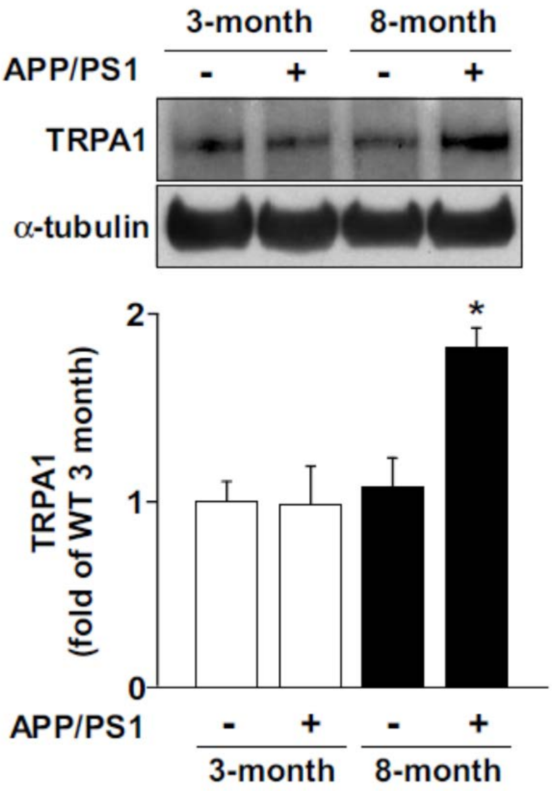

Supplementary Fig. S2. Protein expression of TRPA1 channels in APP/PS1 Tg mice at 3 and 8 months old. Brains were harvested from WT and APP/PS1 Tg mice at 3 and 8 months old. Western blot analysis of TRPA1 and α-tubulin protein levels. Data are mean ± SEM from 6 mice in each group. *, P < 0.05 vs. WT mice.

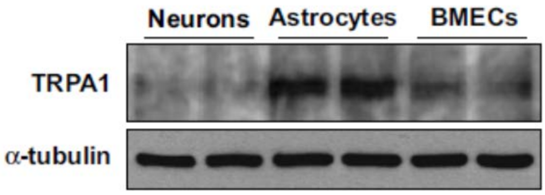

Supplementary Fig. 3. The protein expression of TRPA1 in neurons, astrocytes and endothelial cells. Western blot analysis of TRPA1 and α-tubulin in primary neurons, astrocytes and brain microvascular endothelial cells (BMECs) bEND.3 cells.

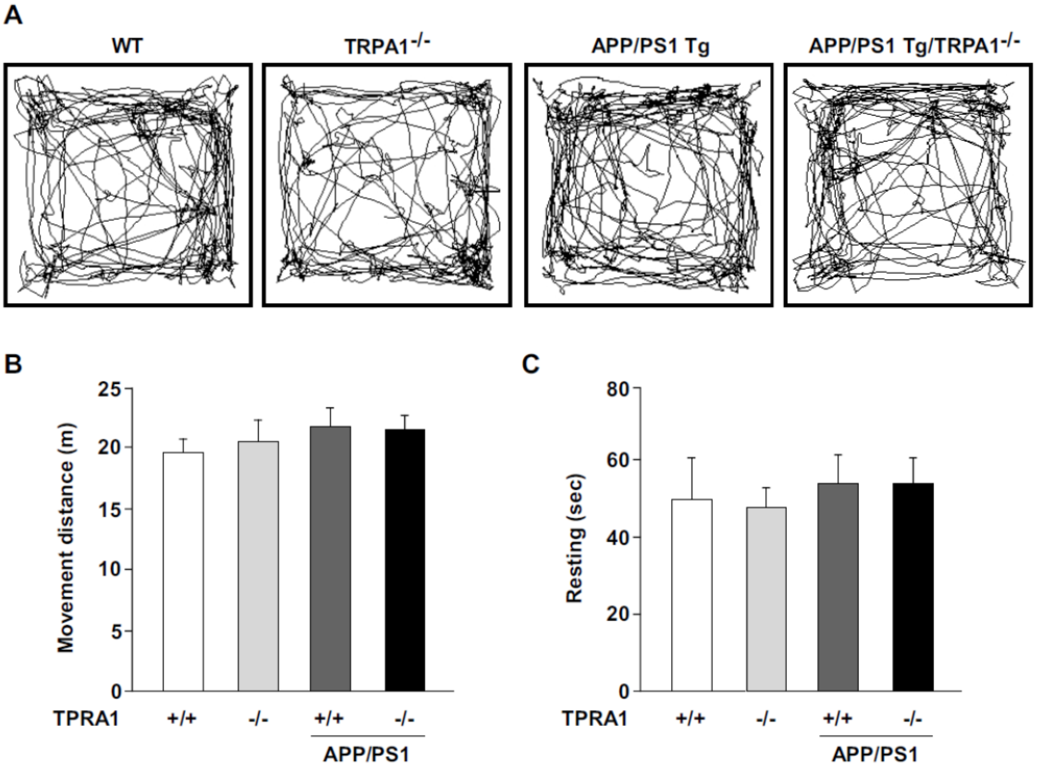

Supplementary Fig. S4. Loss of function of TRPA1 channels does not affect locomotion activity in WT mice, TRPA1$^{-/-}$ mice, APP/PS Tg mice and APP/PS1 Tg/TRPA1$^{-/-}$ mice. (**a**) Representative examples, (**b**) movement distance and (**c**) resting time of open field activity for 8-month-old WT mice, TRPA1$^{-/-}$ mice, APP/PS Tg mice and APP/PS1 Tg/TRPA1$^{-/-}$ mice. Data are mean ± SEM from 8 mice in each group.

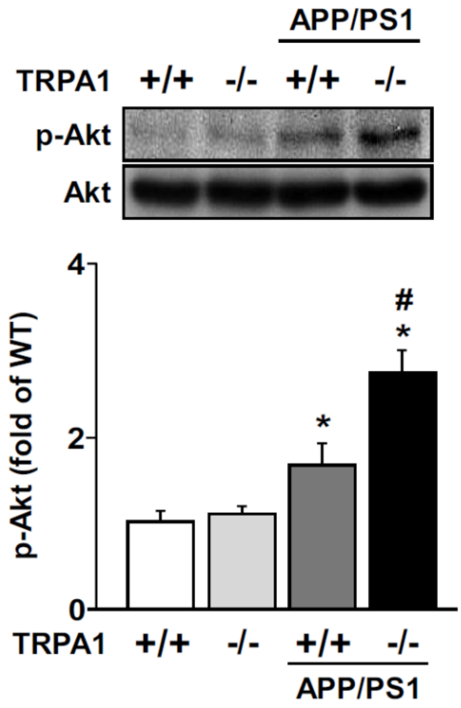

Supplementary Fig. S5. The phosphorylation of Akt is increased in APP/PS1 Tg mice or APP/PS1 Tg/TRPA1$^{-/-}$ mice. Brain specimens were harvested from 8-month-old WT mice, TRPA1$^{-/-}$ mice, APP/PS1 Tg and APP/PS1 Tg/TRPA1$^{-/-}$ mice. Western blot analysis of phosphorylated-Akt and Akt. Data are mean ± SEM from 8 mice in each group. *, $P < 0.05$ vs. WT mice. #, $P < 0.05$ vs. APP/PS1 Tg mice.
